# C89 Induces Autophagy of Female Germline Stem Cells via Inhibition of the PI3K-Akt Pathway In Vitro

**DOI:** 10.3390/cells8060606

**Published:** 2019-06-18

**Authors:** Xinyue Li, Xiaopeng Hu, Geng G. Tian, Ping Cheng, Zezhong Li, Mingyan Zhu, Huchen Zhou, Ji Wu

**Affiliations:** 1 Key Laboratory for the Genetics of Developmental and Neuropsychiatric Disorders (Ministry of Education), Bio-X Institutes, School of Medicine, Shanghai Jiao Tong University, Shanghai 200240, China; lxydyx@sjtu.edu.cn (X.L.); huxiaopeng2017@sjtu.edu.cn (X.H.); gengtian@sjtu.edu.cn (G.G.T.); 2 Key Laboratory of Fertility Preservation and Maintenance of Ministry of Education, Ningxia Medical University, Yinchuan 750004, China; chenping991232@163.com; 3 State Key Laboratory of Microbial Metabolism, School of Pharmacy, Shanghai Jiao Tong University, Shanghai 200240, China; li_zezhong@sjtu.edu.cn (Z.L.); myzhu@sjtu.edu.cn (M.Z.)

**Keywords:** female germline stem cells, compound 89, PI3K-Akt pathway

## Abstract

Postnatal female germline stem cells (FGSCs) are a type of germline stem cell with self-renewal ability and the capacity of differentiation toward oocyte. The proliferation, differentiation, and apoptosis of FGSCs have been researched in recent years, but autophagy in FGSCs has not been explored. This study investigated the effects of the small-molecule compound 89 (C89) on FGSCs and the underlying molecular mechanism in vitro. Cytometry, Cell Counting Kit-8 (CCK8), and 5-ethynyl-2’-deoxyuridine (EdU) assay showed that the number, viability, and proliferation of FGSCs were significantly reduced in C89-treated groups (0.5, 1, and 2 µM) compared with controls. C89 had no impact on FGSC apoptosis or differentiation. However, C89 treatment induced the expression of light chain 3 beta II (LC3BII) and reduced the expression of sequestosome-1 (SQSTM1) in FGSCs, indicating that C89 induced FGSC autophagy. To investigate the mechanism of C89-induced FGSC autophagy, RNA-seq technology was used to compare the transcriptome differences between C89-treated FGSCs and controls. Bioinformatics analysis of the sequencing data indicated a potential involvement of the phosphatidylinositol 3 kinase and kinase Akt (PI3K-Akt) pathway in the effects of C89′s induction of autophagy in FGSCs. Western blot confirmed that levels of p-PI3K and p-Akt were significantly reduced in the C89- or LY294002 (PI3K inhibitor)-treated groups compared with controls. Moreover, we found cooperative functions of C89 and LY294002 in inducing FGSC autophagy through suppressing the PI3K-Akt pathway. Taken together, this research demonstrates that C89 can reduce the number, viability, and proliferation of FGSCs by inducing autophagy. Furthermore, C89 induced FGSC autophagy by inhibiting the activity of PI3K and Akt. The PI3K-Akt pathway may be a target to regulate FGSC proliferation and death.

## 1. Introduction

Female germline stem cell (FGSC) lines were successfully established from postnatal mammal and human ovarian tissue in vitro, and the establishment of these lines has opened up new avenues for research [1,2,3,4]. FGSCs have offered important opportunities for delaying menopause, understanding human oogenesis, and providing a new strategy for preserving fertility [2,5,6,7]. However, the biological activities and regulatory mechanisms of FGSCs, including in proliferation, differentiation, and apoptosis, are less well understood [8,9,10], and there has been no research about the autophagy of FGSCs.

Autophagy is the cellular process of degrading damaged or superfluous organelles and proteins [11,12]. Under normal conditions, autophagy occurs constitutively at a basal level and mainly provides housekeeping functions to maintain cellular function. However, under stress conditions (e.g., during starvation), autophagy is induced to regenerate nutrients or induce cell death [13,14]. Therefore, autophagy can affect the growing status of cells, regulate cell proliferation, or induce cell death. Recent studies have shown that autophagy also plays important roles in both physiological and pathological processes [15,16,17,18,19]. Autophagy is applied to the treatment of many diseases, such as cancer, metabolic disorders, neurodegenerative diseases, and aging through its effects on the growth of related stem cells [20,21,22,23,24]. The phosphatidylinositol 3 kinase and kinase Akt (PI3K-Akt) and adenosine-monophosphate-activated protein kinase-tuberous sclerosis complex (AMPK-TSC) pathways function as upstream signaling pathways of mammalian target of rapamycin (mTOR), a key modulator of the autophagic process. The PI3K-Akt pathway is considered one of the primary pathways regulating autophagy [25].

Chemical compounds show advantages in their application at various concentrations; unlimited structures; typically rapid and easy manipulation; reversible effects; and rapid phenotype-based high-throughput screening [26,27,28]. Many compounds can regulate the progression of autophagy, and have shown significant therapeutic potential in various diseases [29,30]. Some compounds have also been used to regulate the activities of stem cells, including self-renewal, apoptosis, differentiation, and autophagy [8,9,31,32]. Benzoborazoles are widely used in anti-inflammatory, antibacterial, and anti-cancer aspects, and are suitable for clinical and experimental applications. In terms of cell function studies, benzoborazoles are only known to inhibit the growth of human ovarian cancer skov-3 cells, suggesting that they may induce cell autophagy or apoptosis. Compound 89 (C89) is a kind of benzoborazole. C89, 7-formyl-1,3-dihydro-1-hydroxy-2,1-benzoxaborole, was recently synthesized based on the structure of AN2690 [33,34], 5-fluoro-1,3-dihydro-1-hydroxy-2,1-benzoxaborole, an antifungal agent that inactivates fungalleucyl-tRNA synthetase [35]. C89 is a novel compound, and have been no reports about the function of C89 in the autophagy of FGSCs or other kinds of cells.

In this study, we investigated the effects of C89 on FGSCs and the underlying mechanisms. We found that C89 induced autophagy in FGSCs through inhibition of the PI3K-Akt pathway. Based on this, we may find a new approach to promote FGSC survival and proliferation. These findings may help to provide new insights in female infertility.

## 2. Materials and Methods

### 2.1. Animals

Mice (5 days old) were purchased from the SLRC Laboratory Company (Shanghai, China). All animal experiments were approved by the Institutional Animal Care and Use Committee of Shanghai, and were carried out in accordance with the National Research Council Guide for the Care and Use of Laboratory Animals. The ethical approval number is A2016084.

### 2.2. Chemical Compounds

**Figure 1:**
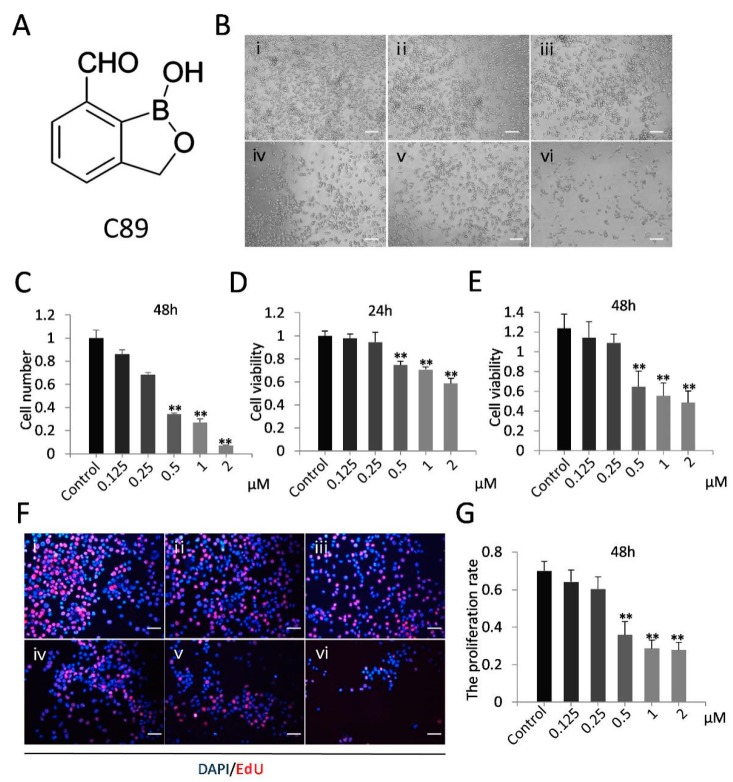
Effect of compound 89 (C89) on female germline stem cell (FGSCs) in vitro. (**A**) Structure of C89. (**B**) Cellular morphology of FGSCs treated with various concentrations of C89at 48 h. Bar: 50µm. i: Control (DMSO), ii: 0.125 μM, iii: 0.25 μM, iv: 0.5 μM, v: 1 μM, vi: 2 μM. (**C**) The amount of various concentrations C89-treated FGSCs. The number of FGSCs was significantly reduced from 0.5to 2µM C89-treated groups compared to control. (**D**,**E**) Cellular viability of FGSCs treated with C89 for 24 h (**D**) or 48 h (**E**) as detected by Cell Counting Kit 8 (CCK8) assay. FGSCs showed significant differences in cell viabilities between the 0.5, 1, and 2 μM C89-treated groups and control groups (*p* < 0.01). (**F**,**G**) Proliferation of C89-treated FGSCs at 48 h as determined using the 5-ethynyl-2’-deoxyuridine (EdU) staining. Significant differences in proliferation were observed between 0.5, 1, and 2 μM C89-treated groups and the control groups (*p* < 0.01). Bar: 25 µm. i: Control (DMSO), ii: 0.125 μM, iii: 0.25 μM, iv: 0.5 μM, v: 1 μM, vi: 2 μM. * *p* < 0.05, ** *p* < 0.01, *** *p* < 0.001.

The synthetic C89 was produced by the laboratory of Professor Huchen Zhou [33,34]. The structure of C89 is shown in Figure 1A. LY294002 (TargetMol Co., Ltd., Shanghai, China) is a specific PI3K inhibitor [36].

### 2.3. Culture of FGSCs In Vitro

The FGSC line was established from mice as described in our previous reports [2,37]. The mouse FGSC line was cultured in vitro according to previously described conditions [4,38]. FGSCs were cultured in Minimum Essential Medium Alpha (Invitrogen, Carlsbad, CA, USA) containing 10% fetal bovine serum (Life Technologies, Carlsbad, CA, USA), 2 mML-glutamine (Amresco, Radnor, PA, USA), 30 mg/mL pyruvate (Amresco), 1 mM nonessential amino acids (Invitrogen Life Sciences, CA, USA), 6 mg/mL penicillin (Amresco), 10 ng/mL mouse basic fibroblast growth factor (PeproTech, London, UK), 10 ng/mL mouse glial cell line-derived neurotrophic factor (PeproTech, NJ, USA), 20 ng/mL mouse epidermal growth factor (PeproTech), 10 ng/mL mouse leukemia inhibitory factor (Santa Cruz Biotechnology), and 50 mM β-mercaptoethanol (Sigma Chemical Co., St. Louis, MO, USA). The SIM-6-thiogunaniaoualiain (STO) cell line (ATCC, Manassas, VA, USA) served as the feeder to culture FGSCs. Cells were passaged every 5 days.

### 2.4. Cell Counting Kit 8 and 5-Ethynyl-2’-Deoxyuridine Labeling Assay

FGSCs (5000 cells) were seeded into a 96-well plate and incubated with different concentrations of C89 (0.125, 0.25, 0.5, 1, 2 μM) for 24 h and 48 h. DMSO (1 μM, Sigma-Aldrich) was used as control. After treatment, Cell Counting Kit 8 (CCK8) solution (10 μL) (Genomeditech, Co., Ltd., Shanghai, China) was added to each well and cells were cultured for 1 h at 37 °C. Absorption values at 450 nm were measured using a Bio-Tek microplate reader (Bio-Tek Instruments, Thermo Fisher Scientific, Winooski, VT, USA). The 5-ethynyl-2’-deoxyuridine (EdU) assay was performed with Cell-Light EdU DNA Cell Proliferation kits (Ribobio, Co., Ltd., Guangzhou, China) used to evaluate cell proliferation according to the manufacturer’s instructions. The cell proliferation index was determined as the ratio of EdU to DAPI and calculated based on the red color of positive cells.

### 2.5. RNA Isolation and Reverse Transcription-Polymerase Chain Reaction

Total RNA was extracted from FGSCs and mouse oocytes using Trizol reagent (Life Technologies, CA, USA) according to the manufacturer’s instructions, and reverse transcription of RNA was performed using the Reverse Transcription Reagent kit (K1622, Fermentas, Hanover, MD, USA) according to the manufacturer’s instructions. The cDNA was stored at −20 °C for further use. All primers used for RNA isolation and reverse transcription-polymerase chain reaction (RT-PCR) are listed in Table S1 (Generay Biotech Co., Ltd., Shanghai, China). RT-PCR was performed in a total volume of 20 μL including 10 μL of Premix, 1 μL of cDNA, 0.2 μL of forward primers (10 μM), 0.2 μL of reverse primers (10 μM), and 8 μL of sterile water. The *Gapdh* gene was used for normalization. The reaction conditions consisted of initial denaturing at 95 °C for 5 min, followed by 30 cycles at 95 °C for 30 s, annealing at 55 °C for 30 s, and extension at 72 °C for 30 s, and a final extension at 72 °C for 10 min. PCR products were examined on an agarose gel stained with ethidium bromide.

### 2.6. Quantitative Real-Time Polymerase Chain Reaction

Total RNA (1000 ng) was reverse transcribed. Quantitative real-time-polymerase chain reaction (qRT-PCR) was performed in a total volume of 10 μL containing 5 μL of FastStart Universal SYBR Green Master (Rox) (Roche Diagnostics, Indianapolis, IN, USA), 1 μL of cDNA, 0.2 μL of forward primers (10 μM), 0.2 μL of reverse primers (10 μM), and 3.6 μL of sterile water. The qRT-PCR conditions were as follows: 95 °C for 30 s, followed by 40 cycles of 95 °C for 5 s and 60 °C for 34 s, and then 95 °C for 15 s, 60 °C for 60 s, and 95 °C for 15 s. Reactions were performed on a 7500 Real-Time PCR System. All qRT-PCR experiments were repeated three times, and gene expression levels were normalized to *Gapdh*. Data analysis was performed by the 2^-ΔΔCt^ method to measure relative expression of the target gene: ΔΔCt = ΔCt experimental group –ΔCt control group, and ΔCt = Ct target gene –Ct *Gapdh*. All primers used for qRT-PCR are listed in Table S1 (Generay Biotech Co., Ltd., Shanghai, China).

### 2.7. Cell Apoptosis Assay

Apoptosis was detected by Annexin V/propidium iodide (PI) (Thermo Fisher Scientific) staining according to the manufacturer’s instructions. Briefly, FGSCs were treated with 0.5 μM C89, collected, washed once with PBS, and resuspended in 1× binding buffer. Cells were incubated with Annexin V for 15 min at room temperature, washed with 1× binding buffer, resuspended, and incubated with PI staining solution for 15 min. Cells were then analyzed by flow cytometry (BD FACSCalibur machine, BD Biosciences, San Jose, CA, USA).

### 2.8. Western Blotting

FGSCs were cultured until they reached 80% confluence and then treated with 0.5 μM C89. Treated FGSCs were lysed with RIPA Lysis Buffer (Yeasen, Co., Ltd., Shanghai, China) containing protease inhibitor (Yeasen, Co., Ltd., Shanghai, China). The bicinchoninic acid (BCA) assay kit (Yeasen, Co., Ltd., Shanghai, China) was used to measure protein concentration. Western blotting was performed as reported with small modifications [39]. Briefly, protein samples (30 μg) were separated by 12% *w*/*v* sodium dodecyl sulfate polyacrylamide gel electrophoresis and transferred onto polyvinylidene fluoride membranes. Membranes were blocked with 5% nonfat milk in tris-buffered saline Tween 20 (TBST) buffer (1 M Tris-HCl, 0.15 M NaCl, and 0.05% Tween-20) at room temperature for 1 h. Membranes were then incubated with primary antibodies against β-tubulin (1:6000, Santa Cruz Biotechnology), LC3B (1:1000, Abcam Inc., Cambridge, MA, USA), SQSTM1 (1:1000, Abcam Inc., Cambridge, MA, USA), PI3K (1:1000, CST Inc., Danvers, MA, USA), phospho-PI3K (p-PI3K) (1:1000, CSTInc., Danvers, MA, USA), Akt (1:1000, CSTInc., Danvers, MA, USA), or phospho-Akt (p-Akt) (1:1000, CSTInc., Danvers, MA, USA) in 5% nonfat milk in TBST buffer at 4 °C overnight. Membranes were then washed three times with TBST for 10 min and incubated with secondary antibodies HRP-conjugated Affinipure Goat Anti-Rabbit IgG (H+L) (1:2000, Proteintech Inc., Wuhan, China) or HRP-Goat Anti-Mouse IgG (H+L) (1:2000, Proteintech Inc., Wuhan, China) in 5% nonfat milk in TBST buffer at room temperature for 1 h. Membranes were washed three times with TBST for 10 min and bands were detected with enhanced chemiluminescence (ECL, Yeasen, Co., Ltd., Shanghai, China). The images were screened by a chemiluminescence imaging system (ProteinSimple, Santa Clara, CA, USA). The densitometry of the bands was analyzed by ImageJ software (v1.52o, NIH, Bethesda, MD, USA). Protein levels were normalized to β-tubulin.

### 2.9. Immunofluorescence

FGSCs were cultured until they reached 80% confluence and then treated with 0.5 μM C89 for 8 h. Immunofluorescence staining was performed according to the previous study with a small modification [40]. Cells were washed in PBS and fixed in pre-cooled methanol for 30 min. Methanol was removed and cells were washed twice with PBS, followed by permeation with 0.5% Triton X-100 for 30 min at room temperature. Cells were washed twice with PBS and blocked with 10% goat serum for 1 h at 37 °C in a wet box. Cells were then incubated with primary antibody against LC3B (1:100, Abcam Inc., Cambridge, MA, USA) in 0.05% BSA at 4 °C overnight. Cells were washed twice with PBS and then incubated with secondary antibodies HRP-conjugated Affinipure Goat Anti-Rabbit IgG (H + L) (1:200, Proteintech Inc., Wuhan, China) in PBS for 1 h at 37 °C. Cells were washed twice with PBS, and nuclei were stained with 4′,6-diamidino-2-phenylindole (1:5000) for 3 min at room temperature. Cells were washed twice with PBS and covered with anti-fluorescence quencher. Cells were visualized and images were obtained with a fluorescence microscope.

### 2.10. RNA Isolation and RNA Sequencing

Total RNA was extracted from FGSCs using Trizol reagent (Life Technologies, CA, USA). Next, 1000 ng of the digested RNA was processed using the VAHTSTM mRNA-seq v2 Library Prep kit for Illumina1 (Vazyme, Co., Ltd., Shanghai, China), and sequencing libraries were created according to the manufacturer’s protocol. Briefly, first- and second-strand cDNA were synthesized using random hexamer primers. The End-It DNA End Repair kit was used to repair the cDNA fragments. An “A” nucleotide was added to the 3′ end of the fragments, followed by adapter ligation. The ligated cDNA products were subjected to PCR amplification. The library quality was determined using a Bioanalyzer 2100 (Agilent, Santa Clara, CA, USA). The Illumina HiSeq 2500 platform (Illumina, San Diego, CA, USA) was used for RNA-seq. The quality of RNA Sequencing (RNA-seq) data was evaluated using FastQC. Raw sequence data have been submitted to the NCBI Sequence Read Archive under accession number GSE128131.

### 2.11. Gene Ontology and Kyoto Encyclopedia of Genes and Genomes Pathway Analysis

Gene Ontology (GO) analysis was performed to elucidate the biochemical process of unique genes of the differentially expressed mRNAs. Kyoto Encyclopedia of Genes and Genomes (KEGG) pathway analysis was applied to identify the significant pathway of the differentially expressed mRNAs. We uploaded the data of differentially expressed mRNAs to DAVID (http://david.abcc.ncifcrf.gov/home.jsp). We obtained the enrichment results. Fisher’s exact test was used to identify the significant results, and the false discovery rate (FDR) was applied to correct the *p*-values (*p* value < 0.05; fold change > 1.5).

### 2.12. Statistical Analyses

All experiments were replicated at least three times for each group. Data are presented as mean ± SEM. Data were analyzed with ANOVA followed by the Fisher’s least significant difference test with SPSS software (Version 22.0; SPSS, Inc., Chicago, IL, USA). Differences were considered significant at *p* < 0.05.

## 3. Results

### 3.1. C89 Reduced Cell Number and Inhibited Cell Viability and Proliferation of FGSCs In Vitro

We first investigated the effects of C89 on FGSCs in vitro. FGSCs were treated with various concentrations of C89 (0.125, 0.25, 0.5, 1, and 2 µM) for 24 and 48 h, and the numbers of viable FGSCs after treatment were counted by a hemocytometer. The number of viable cells was significantly reduced in FGSCs treated with 0.5, 1, and 2 µM C89 compared with controls (Figure 1A–C). We also performed CCK8 and EdU assays to respectively evaluate the effect of C89 on the viability and proliferation of FGSCs. The CCK8 assay showed that the viability of FGSCs was significantly reduced in the 0.5, 1, and 2 μM C89-treated groups at 24 and 48 h compared with controls (Figure 1D). EdU assays showed that the proliferation rate of FGSCs was significantly reduced in 0.5, 1, and 2 μM C89-treated groups at 48 h compared with controls (Figure 1E). These results indicated that certain concentrations (0.5, 1, and 2 μM) of C89 reduced the viability and proliferation of FGSCs in vitro. We selected 0.5 μM C89 for the following experiments.

### 3.2. C89 Induced Autophagy but Not Apoptosis or Differentiation of FGSCs In Vitro

To elucidate the mechanism underlying the decrease in the number of FGSCs after C89 treatment, we next examined the differentiation, apoptosis, and autophagy of C89-treated FGSCs in vitro. We first evaluated the expression of differentiation marker genes *Stra8* and *Sycp3* using RT-PCR. However, no expression of *Stra8* and *Sycp3* was detected in FGSCs treated with C89 for 24 and 48 h or in controls (Figure S1A). These results indicate that C89 does not induce FGSC differentiation in vitro. We also evaluated whether C89 induced FGSC apoptosis by Annexin V/PI staining. However, as shown in Figure S1B, there was no significant difference of apoptotic rate between C89-treated groups and control groups.

**Figure 2:**
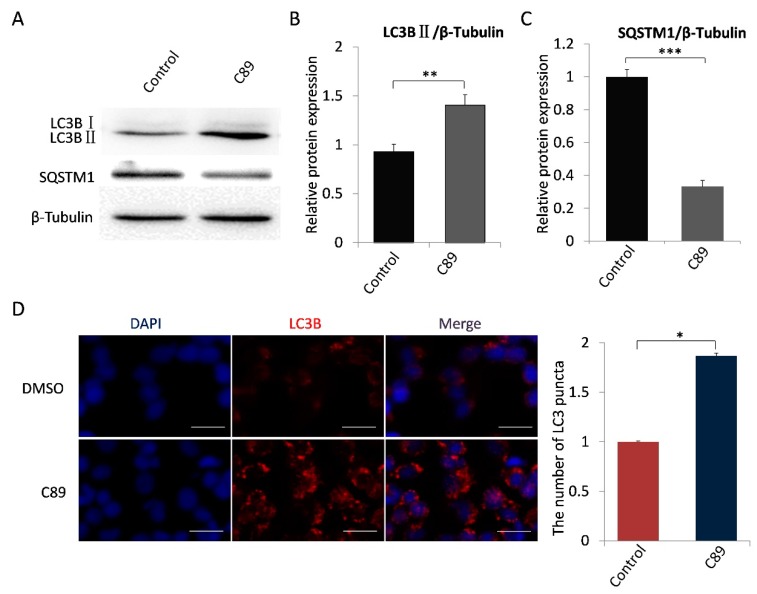
C89 induces autophagy in FGSCs in vitro. (**A**–**C**) Western blot analysis of autophagy marker proteins LC3BII and SQSTM1 in FGSCs treated with C89 or controls. LC3BII expression was significantly higher in C89-treated groups than in control groups (**B**) (*p* < 0.01), and SQSTM1 expression was significantly lower in C89-treated groups than in control groups (**C**) (p < 0.001). (**D**) Immunofluorescence assay of LC3B puncta in C89-treated FGSCs. The number of LC3B puncta per cell in C89-treated groups was significantly higher than in control groups (*p* < 0.01). * *p* < 0.05, ** *p* < 0.01, *** *p* < 0.001.

We next examined whether C89 induced FGSC autophagy. FGSCs were treated with C89 for 8 h, and the expression levels of LC3BII and SQSTM1 were detected by Western blot. As shown in Figure 2A–C, LC3BII expression was significantly higher in C89-treated cells compared with controls, while SQSTM1 expression was significantly lower in C89-treated cells compared with controls. Immunofluorescence was performed to examine the accumulation of LC3B puncta in cells, which represent autophagic vacuoles. The accumulation of LC3B puncta per cell in the C89-treated group was significantly higher than control groups (Figure 2C). Taken together, these results indicate that C89 does not induce FGSC apoptosis or differentiation, but does induce FGSC autophagy in vitro.

### 3.3. Autophagy-Associated Genes and Pathways in C89-Treated FGSCs

**Figure 3:**
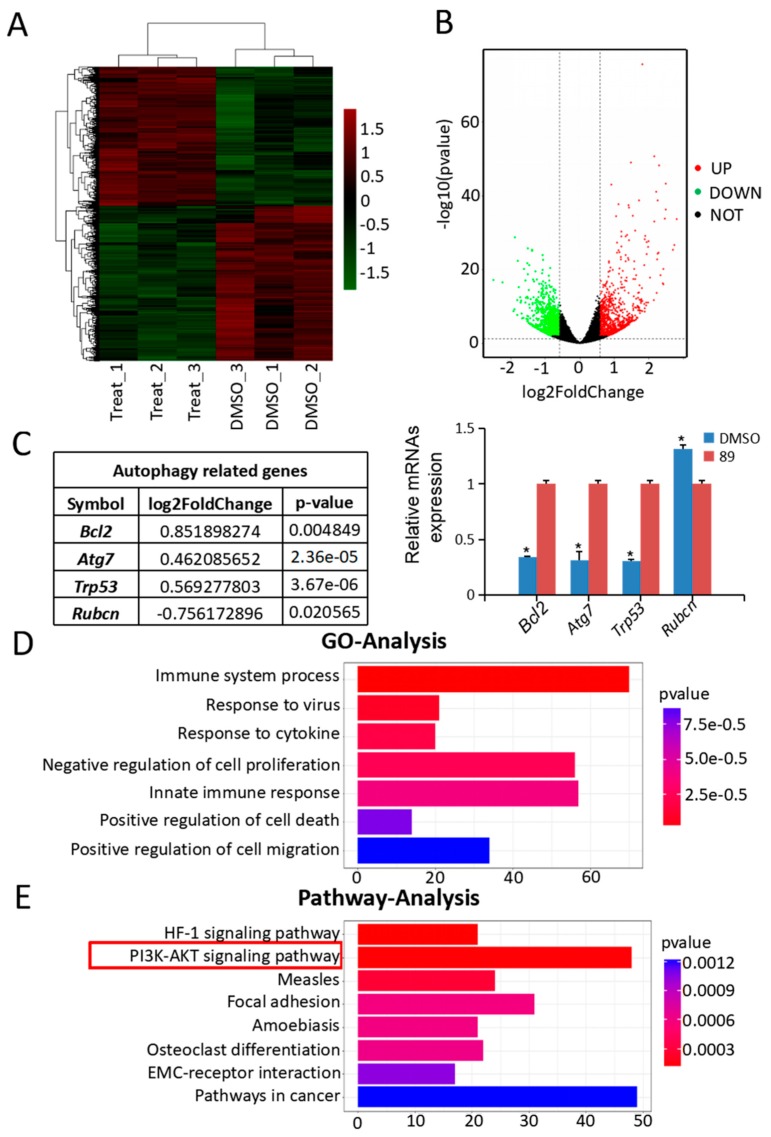
Analysis and validation of RNA-seq data. (**A**) Hierarchical clustering shows differentially expressed mRNA patterns between the control and C89-treated groups. (**B**) Volcano plot of differentially expressed mRNAs. A total of 1983 differentially expressed mRNAs were identified, including 937 upregulated and 1046 downregulated mRNAs. (**C**) Differentially expressed mRNAs were related to autophagy, and the qRT-PCR results were consistent with the changes in transcriptome profile (*p* < 0.05). (**D**) Gene Ontology (GO) analysis of RNA-seq data. The top seven significantly enriched biological processes are shown. (**E**) Kyoto Encyclopedia of Genes and Genomes (KEGG) pathway analysis of RNA-seq data. The top eight enriched pathways are shown. * *p* < 0.05, ** *p* < 0.01, *** *p* < 0.001.

To elucidate the mechanisms underlying C89′s induction of autophagy in FGSCs, we next performed RNA-seq of FGSCs after treatment with 0.5 μMC89 for 8 h. There were three replicates in the C89-treated and control groups, respectively. The RNA-seq data were analyzed by bioinformatics methods. We performed Fast-QC to control the data quality. We required a Q-score higher than 10 (error rate  <  10%) for each replicate (Figure S2A). Moreover, the correlation coefficient of each replicated sample showed very good consistency (Figure S2B). We then analyzed the RNA-seq data after quality screening. After applying a stringent filtering approach that compared control and C89-treated groups (adjusted *p*-value < 0.05; fold change > 1.5), we identified 937 upregulated and 1046 downregulated mRNAs. A heat map (Figure 3A) and a volcano plot (Figure 3B) of the differentially expressed mRNAs were generated by MeV_4_9_0 cluster analysis. Hierarchical clustering of differentially expressed mRNAs showed a clear distinction between the control groups and C89-treated groups. Four of the differentially expressed mRNAs-*Bcl2*, *Atg7*, *Trp53* and *Rubcn*-were related to autophagy. The expressions of these mRNAs were verified by qRT-PCR, and the qRT-PCR results were consistent with RNA-seq data (Figure 3C). Moreover, we found that there were no significant differences in the expression of the differentiation-related genes (*Stra8*, *Sycp3*, *Sycp2*, *Sycp1*, *Syce1,* and *Dmc1*) and the apoptosis-related genes (*Fasl*, *Casp1*, *Cox6b2, Apaf1*, *Atm*, and *Casp8*) between C89-treated and control groups (Figure S3).

GO analysis was used to identify the functional categories of these differentially expressed mRNAs. A total of 220 GO terms in the category of biological processes were significantly enriched at an FDR threshold of < 0.05. The top seven significantly enriched biological processes were immune system process, response to virus, response to cytokine, negative regulation of cell proliferation, innate immune response, positive regulation of cell death, and positive regulation of cell migration (Figure 3D). The most abundant categories were related to growth processes that may inhibit cell proliferation and promote cell death. This result is consistent with the in vitro data showing that C89 reduced the number, viability, and proliferation of FGSCs. KEGG pathway analysis revealed that the top eight enriched pathways were the HIF-1 signaling pathway, PI3K-Akt signaling pathway, measles, focal adhesion, amoebiasis, osteoclast differentiation, ECM-receptor interaction, and pathways in cancer (Figure 3E). Previous studies have shown that the PI3K-Akt pathway is associated with autophagy [25,41,42]. Therefore, we speculated that C89 may induce autophagy in FGSCs via the PI3K-Akt pathway.

### 3.4. C89 Induced FGSC Autophagy by Inhibiting the Phosphorylation of PI3K and Akt

**Figure 4:**
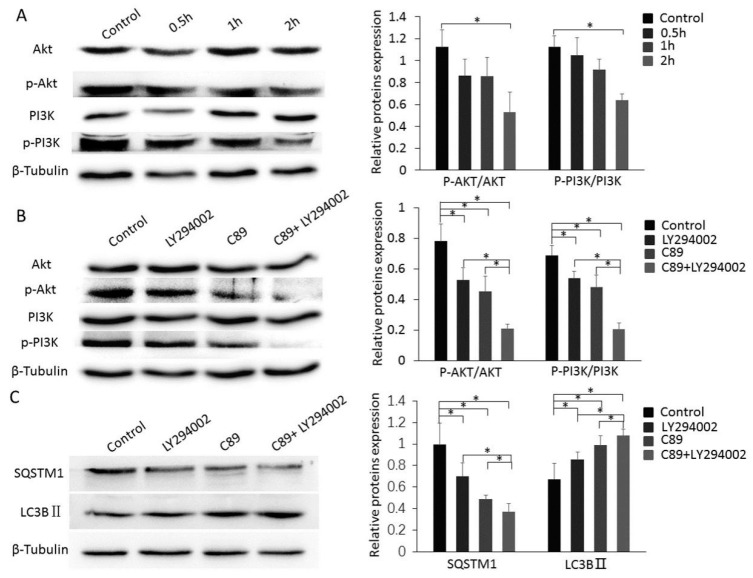
Cooperative functions of C89 and LY294002 in inducing autophagy via suppressing the PI3K-Akt pathway. (**A**) Western blot of Akt, p-Akt, PI3K, and p-PI3K in FGSCs treated with C89. C89 significantly reduced the expression levels of p-Akt and p-PI3K at 2 h (*p* < 0.05). (**B**) C89 and LY294002 reduced the expression levels of p-Akt and p-PI3K. Western blot of Akt, p-Akt, PI3K and p-PI3K in FGSCs treated with LY294002, C89, C89 + LY294002 or controls for 2 h. The expression levels of p-Akt and p-PI3K were significantly lower in LY294002, C89, and C89 + LY294002 groups compared with controls (*p* < 0.05). Levels of p-PI3K and p-Akt were significantly lower in the LY294002 + C89-treated group compared with LY294002- or C89-treated groups (*p* < 0.05). (**C**) C89 and LY294002 induced FGSC autophagy. The expressions of SQSTM1 were significantly lower, and the expressions of LC3BII were significantly higher in LY294002, C89, and C89 + LY294002 groups compared with control groups (*p* < 0.05). SQSTM1 expression was significantly lower and LC3BII expression was higher in LY294002 + C89-treated cells than in LY294002- or C89-treated cells (*p* < 0.05). * *p* < 0.05, ** *p* < 0.01, *** *p* < 0.001.

To evaluate the potential role of the PI3K-Akt pathway in C89-induced FGSC autophagy, we examined the expression levels of PI3K, p-PI3K, Akt, and p-Akt in FGSCs after 0.5, 1, and 2 h treatment with C89. Both p-PI3K and p-Akt levels were significantly lower in FGSCs treated with C89 for 2 h compared with controls (Figure 4A). These results suggest that C89 inhibits the activation of PI3K and Akt in FGSCs, indicating that the PI3K-Akt pathway is inhibited by C89 in FGSCs.

### 3.5. Cooperative Functions of C89 and LY294002 in Inducing Autophagy via Suppressing the PI3K-Akt Pathway

To further clarify the role of C89 in inhibiting PI3K and Akt activity and promoting FGSC autophagy, we used the PI3K inhibitor LY294002. FGSCs were treated with LY294002 (5 µM), C89 (0.5 µM), LY294002 (5 µM) + C89 (0.5 µM), or DMSO (control) for 2 or 8 h. The expression levels of p-PI3K, p-Akt, LC3BII, and SQSTM1 were detected by Western blot. The expression levels of p-PI3K, p-Akt, and SQSTM1 in LY294002- or C89-treated FGSCs were significantly lower than in controls. Furthermore, expression levels of p-PI3K, p-Akt, and SQSTM1 in FGSCs treated with LY294002 + C89 were significantly lower than in cells treated with either single treatment. The expression levels of LC3BII showed a similar trend and were significantly higher in singly-treated cells compared with controls, while LC3BII levels were significantly higher in FGSCs treated with LY294002 + C89 compared with single treatments (Figure 4B,C). These results suggest that both C89 and LY294002 induced FGSC autophagy by inhibiting the activity of Akt and PI3K. These results also suggest that C89 and LY294002 cooperatively inhibited PI3K-Akt pathway activity and enhanced FGSC autophagy.

## 4. Discussion

The development of FGSCs plays an important role in the maintenance of ovarian function. Although a few studies have revealed the molecular mechanisms of proliferation, differentiation, and apoptosis in FGSCs [8,9,10], no studies have reported autophagy in FGSCs. In this report, we found that the synthetic compound C89 induced FGSC autophagy by inhibiting the activity of Akt and PI3K.

Autophagy is the cellular process of degrading cytoplasmic proteins or organelles [43]. Previous studies have shown that autophagy is crucial for maintaining stemness in various stem cells, and autophagy is applied to the clinical treatment of many stem-cell-related diseases, including neurodegenerative disease and cancer [22,29]. Under certain conditions, autophagy can lead to autophagic programmed cell death, and the number of cells would decrease [44]. Studying autophagy will be beneficial for the clinical application of FGSCs. Our results showed that the proliferation and viability of FGSCs in vitro were significantly decreased by C89 in a dose-dependent manner. Furthermore, C89 induced FGSC autophagy by changing the expression of autophagy marker proteins such as LC3BIIand SQSTM1 [12]. These findings indicate that the synthetic compound C89 can induce FGSC autophagy, resulting in a decrease in the number of FGSCs.

Several studies have demonstrated that the PI3K-Akt pathway regulates autophagy in stem cells [25,41]. Furthermore, the autophagy of deviant stem cells mediated by the PI3K-Akt pathway can cause disease such as neurodegenerative damage, inflammation, and cancer [45,46,47]. The PI3K-Akt pathway plays an important role in regulating cell death and proliferation, and is a target in the treatment of many diseases [48]. To elucidate the mechanism underlying the effect of C89′s induction of autophagy in FGSCs, we used RNA-seq technology to compare differentially expressed mRNAs in control and C89-treated FGSCs. A total of 1985 differentially expressed mRNAs were identified, including 939 upregulated and 1046 downregulated mRNAs. GO and KEGG analyses of the sequencing data indicated an involvement of the PI3K-Akt pathway in the effects of C89 on FGSCs. Western blot confirmed that p-PI3K and p-Akt levels were significantly decreased in C89-treated FGSCs. These resultssuggest that the PI3K-Akt pathway may be a potential target for activating the appropriate level of autophagy to protect FGSCs from aging, and inhibiting autophagy to treat the reproductive disease of excessive autophagy in FGSCs.

To further confirm the function of the PI3K-Akt pathway, the specific PI3K inhibitor LY294002 was used. Both C89 and LY294002 inhibited the activity of p-Akt and p-PI3K to induce FGSC autophagy. Additionally, FGSC autophagy was remarkably enhanced by co-treatment of C89 and LY294002 compared with single treatments. LY294002 is used in many experimental studies as an inhibitor of PI3K [49,50]. C89 is a novel compound synthesized based on the structure of AN2690 [34], but the effects and molecular mechanism of C89 in cells has been unreported. Our findings indicate that C89 may be used as a potential novel inhibitor of the PI3K-Akt pathway in experimental researches and clinical applications.

In conclusion, our study demonstrates that C89 can induce FGSC autophagy in vitro, leading to a decrease in the number of FGSCs. RNA-seq and Western blot demonstrated that C89 induces autophagy in FGSCs via the PI3K-Akt pathway. We also showed that C89 and LY294002 cooperatively inhibited PI3K-Akt pathway activity and enhanced FGSC autophagy.In further research, we will conduct in-depth studies on the molecular mechanisms of C89-induced FGSC autophagy involving the PI3K-Akt pathway to identify more drug screening targets for clinical application. We will also investigate whether C89 induces autophagy in other kinds of cells of ovary and testis, and whether it can be applied to the treatment of clinical autophagy-related diseases.

## Supplementary Materials

Supplementary materials can be accessed at https://www.mdpi.com/2073-4409/8/6/606/s1. Figure S1: C89 had no effect on differentiation and apoptosis of FGSCs at 24 h or 48 h in vitro.; Figure S2: RNA-Seq quality control.; Figure S3: RNA-Seq results of some differentiation- and apoptosis-related genes.; Table S1: The sequence of primers used for PCR.
