# Supplementary material for: C89 Induces Autophagy of Female Germline Stem Cells via Inhibition of the PI3K-Akt Pathway In Vitro
Source: Cells. 2019 Jun 18;8(6):606. doi: 10.3390/cells8060606 (PMC6627605; doi:10.3390/cells8060606)
Supplement: Supplementary file 1 [file cells-08-00606-s001.pdf]

Supplementary Matrials

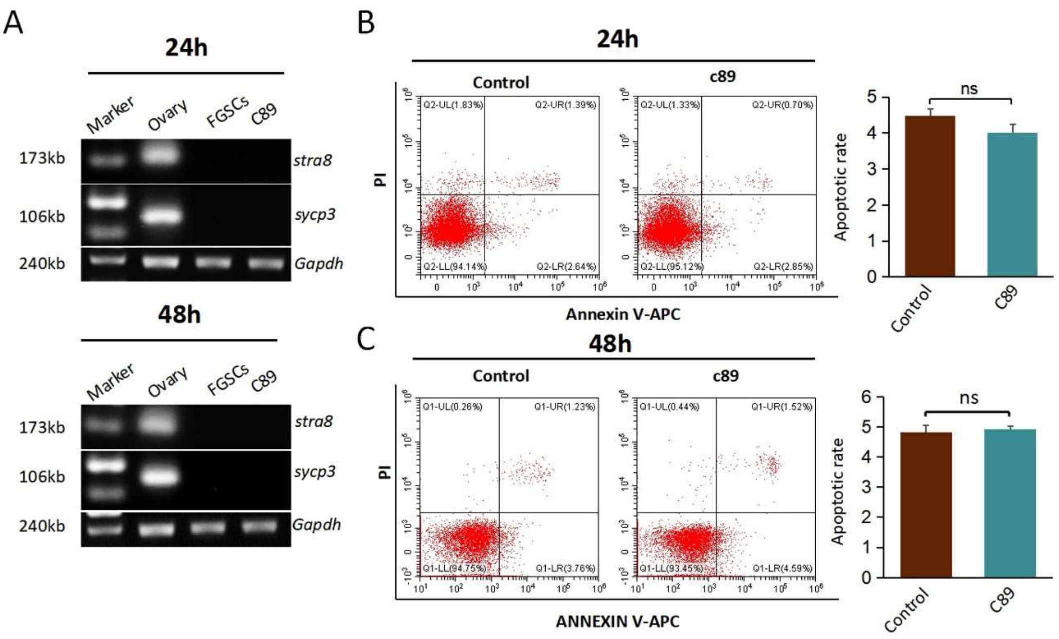

Figure 1. C89 had no effect on differentiation and apoptosis of FGSCs at 24 h or 48 h in vitro. (**A**) The expression of differentiation markers of FGSCs was detected by RT-PCR at 24 h and 48 h. No expressions of *Sycp3* and *Stra8* were detected in FGSCs and C89-treated FGSCs. Ovary served as the positive control. (**B**–) FGSC apoptosis was detected by flow cytometry. The apoptotic rate showed no significant difference between C89-treated groups and control groups at 24 h (**B**) or 48 h (**C**). ns: not significant.

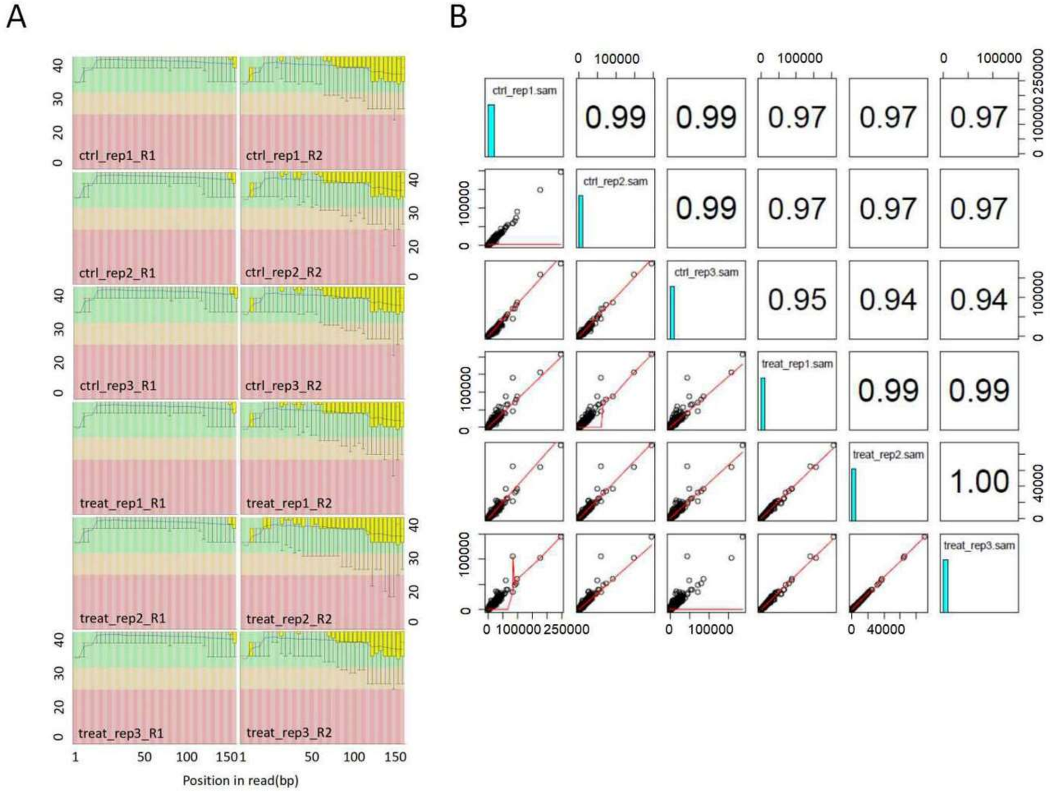

Figure 2. RNA-Seq quality control. (**A**) The Fast-QC data showing the position-specific sequencing quality in each replicated group. Data filtering criteria: Q-score higher than 10 (error rate < 10%). (**B**) The correlation plots of each replicated sample. The correlation coefficient of each replicated sample showed very good consistency. Ctrl: DMSO-treated group, treat: C89-treated group.

A

Differentiation related genes			
Symbol	BaseMean	Log2FoldChange	P-value
Stra8	0	NA	NA
Sycp3	0.821181976	-0.138526804	NA
Sycp2	0	NA	NA
Sycp1	44.15313238	0.209594478	0.626644766
Syce1	0	NA	NA
Dmc1	0.856228319	0.06892028	NA

B

Apoptosis related genes			
Symbol	BaseMean	Log2FoldChange	P-value
Fasl	0.398275911	-0.046778161	NA
Casp1	0	NA	NA
Cox6b2	2.327389915	0.251237924	NA
Apaf1	716.1501603	-0.153586386	0.304142427
Atm	1217.959625	-0.121556373	0.601505372
Casp8	1215.923173	0.103682703	0.539648625

Figure 3. RNA-Seq results of some differentiation- and apoptosis-related genes. (**A**) The fragments per kilobase million (FRPK) of some differentiation-related genes. (**B**) The FRPK of some apoptosis related genes. NA, not available.

Table S1: the sequence of primes used for PCR

PCR	Forward (5'-3')	Reverse (5'-3')
Gapdh	GTCGTGGAGTCTACTGGTGTC	GAGCCCTTCCACAATGCCAAA
Stra8	ACAACCTAAGGAAGGCAGTTTAC	GACCTCCTCTAAGCTGTTGGG
Sycp3	AGCCAGTAACCAGAAAATTGAGC	CCACTGCTGCAACACATTCATA
Bcl2	CATGCCGTCCGAGAAGACCT	GATGAGCCGGACATCTTCCACT
Trp53	GGAATCTCACCCCATCCCA	CAGTAAGCCAAGATCACGCC
ATG7	GCCTATATGTACTGCTTCATCCA	CATTTCAGGGGTGTGCCTTCA
Rubicon	AACCTCACCCACCATCTTCTTAGCG	CACAGAGTTAAGTGCATAATTGGCATAAAG
